# Cellular plasticity of pathogenic fungi during infection

**DOI:** 10.1371/journal.ppat.1008571

**Published:** 2020-06-04

**Authors:** Kenya E. Fernandes, Dee A. Carter

**Affiliations:** School of Life and Environmental Sciences and the Marie Bashir Institute for Infectious Diseases and Biosecurity, University of Sydney, Sydney, Australia; University of Maryland, Baltimore, UNITED STATES

## Introduction

A widespread trait amongst fungi is their ability to alter their morphology in response to environmental stimuli. The type and degree of alteration, which commonly includes changes in cell size and shape, can vary between strains and even between individual cells within genetically uniform populations, providing many levels of variation within species. As well as enhancing their ability to survive in different environmental niches, this variation plays an important role in the ability of human fungal pathogens to survive and cause disease in the host. Some well-known examples are the thermally dimorphic fungi, which grow as moulds at 22 °C–25 °C in the soil and covert into yeasts at 37 °C when in a mammalian host [1]. Some fungi, rather than switching between 2 distinct forms in different niches, produce a variety of forms in the host that appear to have roles in the infection process. This phenomenon has not been as extensively studied as thermal dimorphism but may be important for understanding disease progression and outcome. Here, we review selected examples of pathogenic fungi that produce distinct morphological forms when living in and on host tissues that may be linked to infection and virulence. Two genera, *Candida* and *Malassezia*, are highly adapted to the commensal lifestyle and can also become pathogenic, and 2 others, *Coccidioides* and *Cryptococcus*, have environmental components to their life cycle but show evidence of adaptation to life inside a host.

## White-to-opaque and yeast-to-hyphae transitions in *Candida albicans*

**Fig 1:**
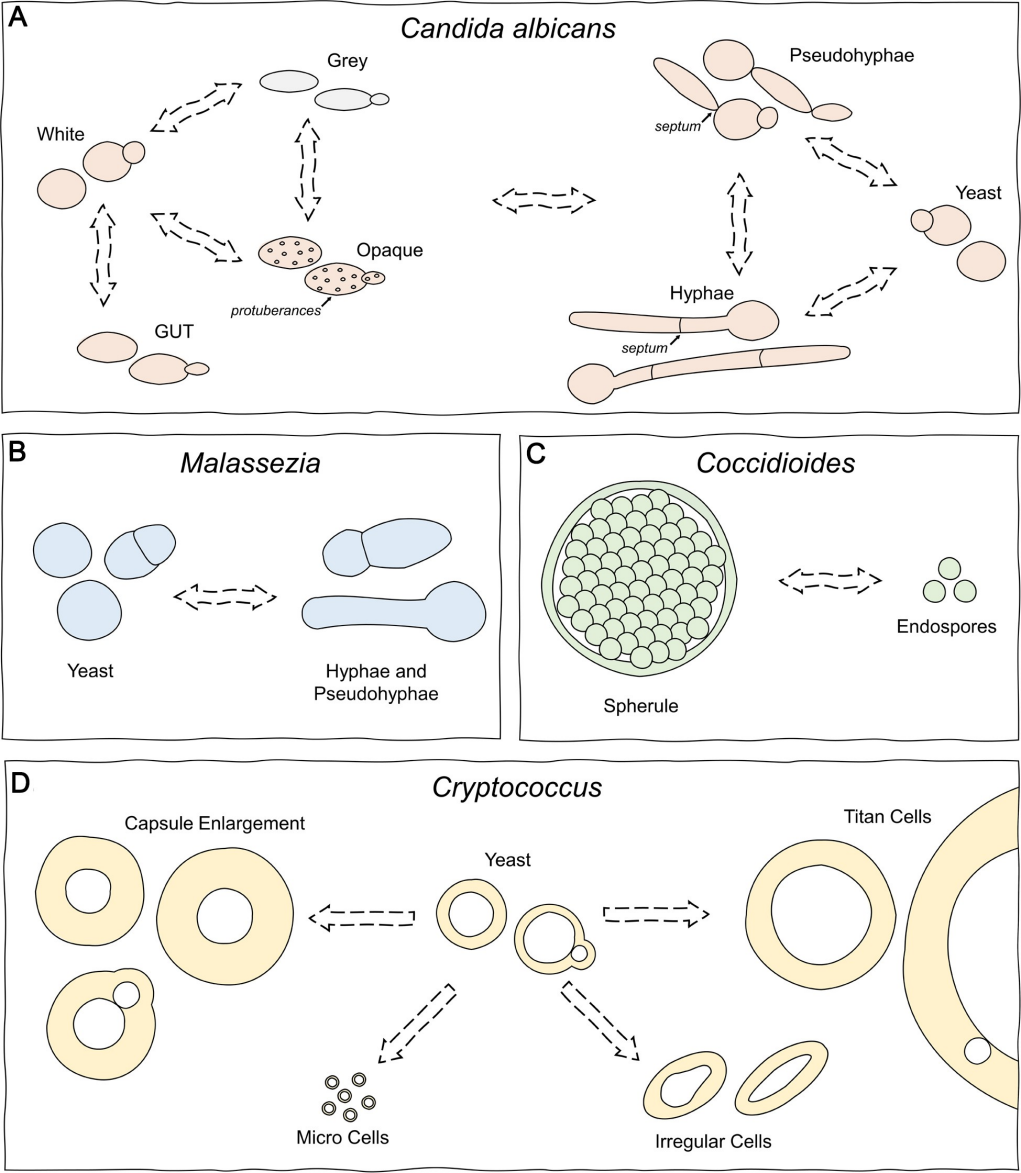
Morphological switches and transitions during the fungal infection process. (A) Yeast-to-hyphae-to-pseudohyphae transformations and switching between white, grey, opaque, and GUT yeast forms in *C*. *albicans*. (B) Reversible yeast-hyphae switching in *Malassezia*. (C) The spherule-endospore cycle, unique to *Coccidioides* infections. (D) Differentiation of regular yeast cells to produce various morphologically different forms in *Cryptococcus*. GUT, gastrointestional-induced transition.

*C*. *albicans* is a commensal of humans that colonises the mucosal surfaces of most healthy individuals but can cause life-threatening infections in immunocompromised hosts [2]. The ability to thrive in different niches within the host is crucial for survival, and *C*. *albicans* possesses an array of morphological forms that are thought to aid it in this process (Table 1; Fig 1A). During commensal growth, *C*. *albicans* exhibits a range of morphologies that appear to be suited to various host niches [3]. White, opaque, grey, and gastrointestinal-induced transition (GUT) yeast cell types have been described, and *C*. *albicans* can switch between these, enabling it to adapt rapidly to changes in its environment. White cells are smooth and round, whereas opaque cells are elongated, with more vacuoles and cell surface protuberances, and grey cells are the smallest cell type and are elongated with no protuberances [4, 5]. Opaque cells have been found to mate more efficiently than white cells and, along with grey cells, are more virulent and capable of faster proliferation on epithelial surfaces, whilst white cells are more virulent in systemic bloodstream infection models [6–8]. Opaque cells also possess increased resistance to macrophages and neutrophils because, unlike white cells, they do not secrete a chemoattractant, and it has been suggested that the switch to opaque cells may be a mechanism to escape the immune system [9]. GUT cells are morphologically similar to opaque cells but do not possess cell surface protuberances, are unable to mate, and seem to be specialised for commensalism in the mammalian gastrointestinal tract, displaying superior fitness to other cell types in models of this niche [10].

**Table 1:** The variety of morphological forms produced by pathogenic fungi that may play a role during the infection process.

Fungus	Phylum	Morphological Form	Description
*C*. *albicans*	Ascomycota	hyphae	long, tube-shaped filaments, multicellular
		pseudohyphae	elongated ellipsoids, multicellular
		white cells	small, round-to-oval
		grey cells	small, elongated ovals
		opaque cells	larger elongated ovals, more vacuoles, surface protuberances
		GUT cells	larger elongated ovals
*Malassezia* spp.	Basidiomycota	hyphae	elongated, 10–25 μm in length
		regular yeasts	round, 8 μm in diameter
		ovoid cells*	ovoid, 2.5–6 μm in length
		cylindrical cells*	short variants 1.5–3 μm in length, long variants 6 μm in length
*Coccidioides immitis* and *C*. *posadasii*	Ascomycota	spherules	spherical, 30–80 μm in diameter, containing 100–300 endospores
		endospores	spherical, 2–7 μm in diameter
		arthroconidia*	can be highly variable, including spherical, triangular, and barrel-shaped
		fungal ball*	spheroid mass of highly branched hyphae
*Cryptococcus neoformans*/*gattii* complex	Basidiomycota	regular yeasts	spherical, 4–7 μm in diameter
		capsule enlargement	cells with large polysaccharide capsules
		titan cells	greater than 15 μm in diameter, thickened cell walls, larger vacuoles
		micro cells	smaller than 1 μm in diameter
		irregular cells*	elongated ellipsoids or tapered and egg-shaped
		pseudohyphae*	elongated ellipsoids, multicellular

*Less well-characterised morphological forms with currently unknown implications for virulence.

**Abbreviations:** GUT, gastrointestinal-induced transition.

With host immunosuppression, *C*. *albicans* can become an opportunistic pathogen, and this is accompanied by transitions from the unicellular yeast form to hyphal and pseudohyphal cell types [3]. *Candida* hyphae are long, filamentous, and multicellular [11], whilst pseudohyphae are elongated ovals that have features of both yeasts and hyphae. Depending on environmental factors, these 3 cell types can either stably proliferate to maintain their cell type or transition to the other cell types, and the ability to do so is a crucial determinant of virulence in *C*. *albicans*. Hyphal and pseudohyphal forms are invasive and are thought to have an increased ability to penetrate into host tissues and internal organs and cause damage, whilst yeast cells may aid dissemination through the bloodstream because of their small size [15, 16]. Mutants that cannot interchange between these cell types are typically defective in infection models, and isolates taken from patients with disseminated candidiasis generally contain both yeast and hyphal forms [12, 13]. Biofilms, which are complex and densely packed communities of cells with increased resistance to host defences and antifungal drugs, also typically contain all 3 cell types [14].

## Yeast-to-hyphae transition and pleomorphism in *Malassezia* spp.

*Malassezia* spp. are lipid-dependent yeasts that are a major component of the normal skin mycobiome of humans and other animals, although metagenomic sequencing studies have found *Malassezia* DNA in various terrestrial and marine habitats, suggesting some species may inhabit a wider ecological niche than was previously assumed [17–19]. Under certain conditions that induce fungal overgrowth or through host immunocompromisation, commensal *Malassezia* species can become opportunistic pathogens, and cause a variety of dermatological conditions. In host tissues, *Malassezia* can reversibly switch between yeasts of around 8 μm in diameter and hyphae or pseudohyphae of 10–25 μm in length (Table 1; Fig 1B), resulting in a characteristic “spaghetti and meatballs” appearance in scrapings from infected skin or lesions [20]. The yeast form of *Malassezia* has many known virulence attributes, including the ability to either up-regulate or suppress the immune response [21]. Hyphal forms are difficult to produce in vivo and are therefore less well understood, but it is thought that they can penetrate keratinised skin cells, gaining access to deeper nutrient-rich layers where they can revert to the yeast form and proliferate to replace cells being shed at the epidermal surface [15]. Beyond these commonly known forms, considerable other variations in the morphology of *Malassezia* have been observed, including ovoid and cylindrical cells and hyphae of varying size [22] (Table 1). To date, these morphologies have received little attention, and they may play distinct and potentially important roles in infection.

## Spherule formation and hyphal polymorphism in *Coccidioides immitis* and *C*. *posadasii*

*C*. *immitis* and *C*. *posadasii* are soil-dwelling pathogenic fungi that cause pulmonary infection via airborne arthroconidia, which can develop into life-threatening disseminated disease in at-risk individuals [23]. Although originally thought to be an accidental pathogen and opportunist, recent studies indicate that the infection of small animals such as bats and armadillos may form part of the life cycle of *Coccidioides* and that it has evolved specific adaptations for host interaction [24]. Central to its pathogenicity during human infection is the production of spherules and endospores. Spherules are formed from a gradual enlargement and transformation of inhaled arthroconidia into a structure that is typically 30–80 μm in diameter and contains around 100–300 endospores [25] (Table 1; Fig 1C). Upon rupture of the spherule, the endospores are released into host tissues, where each can produce hyphal growth or develop into a new spherule, repeating the growth cycle [26]. In addition to temperature and CO_2_ levels, there is evidence that the transition to spherules can be stimulated by contact with neutrophils, and reversion of arthroconidia to hyphal forms has occasionally been observed in infected lung cavities with no neutrophils [23]. Other, less well-characterised morphotypes have also been observed clinically, but their role in infection is not well understood (Table 1) [27, 28].

## Variation in capsule and cell size in *Cryptococcus*

*Cryptococcus neoformans* and members of the *C*. *gattii* species complex are encapsulated yeasts that cause severe respiratory and cerebral disease, primarily amongst immunocompromised individuals [29]. Although the pathogenic *Cryptococcus* species are considered to be environmental fungi, adaptations for survival during interactions with environmental predators such as amoebae, insects, and other short-lived organisms are thought to explain their broad host range and possession of various pathogenic traits [30]. *Cryptococcus* cells are typically spherical and 4–7 μm in diameter, but during human infection, the appearance of forms of varying size is common (Table 1; Fig 1D). The polysaccharide capsule possessed by *Cryptococcus* cells is highly dynamic and undergoes substantial enlargement during human infection [31]. Cells with enlarged capsules have been shown to be more resistant to oxidative stress, antimicrobial peptides, and phagocytosis and are generally associated with more severe pathology [32]. Both *C*. *neoformans* and members of the *C*. *gattii* complex can produce highly enlarged “titan” cells, which are greater than 15 μm in diameter and have been seen to reach up to 100 μm. Titan cells have unique characteristics, including thickened cell walls, dense capsules, large vacuoles, and polyploidy [33]. These traits appear to contribute to survival in the host, with their capsular properties increasing resistance to oxidative, nitrosative, and other stresses and their massive size preventing phagocytosis and elimination by macrophages [34]. When replicating, titan cells produce regular-sized progeny; hence, they are always part of a heterogenous mixture of cell types [35].

At the other end of the spectrum, *C*. *neoformans* can produce “micro” cells, a subpopulation of cells that are smaller than 1 μm in diameter [36]. Whilst much less research has been done on micro cells, they are a cell type that has been seen during human infection, and it is thought that they may cross biological barriers more readily because of their small size, aiding dissemination of the pathogen to other body sites [37, 38]. Micro cells are seen in different *C*. *neoformans* varieties and genotypes but have not been observed in species of the *C*. *gattii* complex [39]. Recent studies have further identified cells with unusual, irregular morphologies in some *Cryptococcus* strains. These can be tapered and egg-shaped or elongated and of a more pseudohyphal form. Their presence in clinical populations has been associated with increased antifungal tolerance but decreased virulence, which suggests they may promote persistence in the host [38, 39]. Like titan cells, micro and irregular cell types always appear as part of a mix of different cell types.

## Conclusions

Diverse fungal morphologies are increasingly being recognised as key traits associated with virulence, aiding pathogens in various ways, including adhesion to physiological barriers, dissemination through the body, and manipulation of host immune responses [40]. Whilst some morphological forms have well-characterised implications for virulence and pathogenesis, there is still much to learn about the capacity of fungi for morphogenesis during disease progression. This knowledge may ultimately help inform disease diagnosis and prognosis, with implications for treatment strategies.

## References

[1] Sil A, Andrianopoulos A. Thermally dimorphic human fungal pathogens—polyphyletic pathogens with a convergent pathogenicity trait. Cold Spring Harb Perspect Med. 2014;5(8): a019794 10.1101/cshperspect.a019794 PubMed Central PMCID: PMC4526722. 25384771 PMC4526722

[2] Sardi JC, Scorzoni L, Bernardi T, Fusco-Almeida AM, Mendes Giannini MJ. *Candida* species: current epidemiology, pathogenicity, biofilm formation, natural antifungal products and new therapeutic options. J Med Microbiol. 2013;62(1): 10–24. 10.1099/jmm.0.045054-0 23180477

[3] Noble SM, Gianetti BA, Witchley JN. *Candida albicans* cell-type switching and functional plasticity in the mammalian host. Nat Rev Microbiol. 2017;15(2): 96–108. 10.1038/nrmicro.2016.157 PubMed Central PMCID: PMC5957277. 27867199 PMC5957277

[4] Anderson J, Mihalik R, Soll DR. Ultrastructure and antigenicity of the unique cell wall pimple of the *Candida* opaque phenotype. J Bacteriol. 1990;172(1): 224–35. 10.1128/jb.172.1.224-235.1990 PubMed Central PMCID: PMC208422. 2403540 PMC208422

[5] Bommanavar SB, Gugwad S, Malik N. Phenotypic switch: the enigmatic white-gray-opaque transition system of *Candida albicans*. J Oral Maxillofac Pathol. 2017;21(1): 82–6. 10.4103/0973-029X.203781 PubMed Central PMCID: PMC5406825. 28479692 PMC5406825

[6] Kvaal C, Lachke SA, Srikantha T, Daniels K, McCoy J, Soll DR. Misexpression of the opaque-phase-specific gene *PEP1 (SAP1)* in the white phase of *Candida albicans* confers increased virulence in a mouse model of cutaneous infection. Infect Immun. 1999;67(12): 6652–62. 10569787 10.1128/iai.67.12.6652-6662.1999 PMC97079

[7] Tao L, Du H, Guan G, Dai Y, Nobile CJ, Liang W, et al Discovery of a "white-gray-opaque" tristable phenotypic switching system in *Candida albicans*: roles of non-genetic diversity in host adaptation. PLoS Biol. 2014;12(4): e1001830 10.1371/journal.pbio.1001830 PubMed Central PMCID: PMC3972085. 24691005 PMC3972085

[8] Xie J, Tao L, Nobile CJ, Tong Y, Guan G, Sun Y, et al White-opaque switching in natural MTLa/alpha isolates of *Candida albicans*: evolutionary implications for roles in host adaptation, pathogenesis, and sex. PLoS Biol. 2013;11(3): e1001525 10.1371/journal.pbio.1001525 PubMed Central PMCID: PMC3608550. 23555196 PMC3608550

[9] Sasse C, Hasenberg M, Weyler M, Gunzer M, Morschhauser J. White-opaque switching of *Candida albicans* allows immune evasion in an environment-dependent fashion. Eukaryot Cell. 2013;12(1): 50–8. 10.1128/EC.00266-12 PubMed Central PMCID: PMC3535852. 23125350 PMC3535852

[10] Pande K, Chen C, Noble SM. Passage through the mammalian gut triggers a phenotypic switch that promotes *Candida albicans* commensalism. Nat Genet. 2013;45(9): 1088–91. 10.1038/ng.2710 PubMed Central PMCID: PMC3758371. 23892606 PMC3758371

[11] Sudbery P, Gow N, Berman J. The distinct morphogenic states of *Candida albicans*. Trends Microbiol. 2004;12(7): 317–24. 10.1016/j.tim.2004.05.008 15223059

[12] Di Carlo P, Di Vita G, Guadagnino G, Cocorullo G, D'Arpa F, Salamone G, et al Surgical pathology and the diagnosis of invasive visceral yeast infection: two case reports and literature review. World J Emerg Surg. 2013;8(1): 38 10.1186/1749-7922-8-38 PubMed Central PMCID: PMC3849356. 24067049 PMC3849356

[13] Lo HJ, Kohler JR, DiDomenico B, Loebenberg D, Cacciapuoti A, Fink GR. Nonfilamentous *C*. *albicans* mutants are avirulent. Cell. 1997;90(5): 939–49. 10.1016/s0092-8674(00)80358-x 9298905

[14] Desai JV, Mitchell AP. *Candida albicans* biofilm development and its genetic control. Microbiol Spectr. 2015;3(3): MB-0005–2014. 10.1128/microbiolspec.MB-0005-2014 PubMed Central PMCID: PMC4507287. 26185083 PMC4507287

[15] Brand A. Hyphal growth in human fungal pathogens and its role in virulence. Int J Microbiol. 2012;2012: 517529 10.1155/2012/517529 PubMed Central PMCID: PMC3216317. 22121367 PMC3216317

[16] Mayer FL, Wilson D, Hube B. *Candida albicans* pathogenicity mechanisms. Virulence. 2013;4(2): 119–28. 10.4161/viru.22913 PubMed Central PMCID: PMC3654610. 23302789 PMC3654610

[17] Theelen B, Cafarchia C, Gaitanis G, Bassukas ID, Boekhout T, Dawson TL Jr. *Malassezia* ecology, pathophysiology, and treatment. Med Mycol. 2018;56(suppl_1): S10–S25. 10.1093/mmy/myx134 . 29538738

[18] Amend A. From dandruff to deep-sea vents: *Malassezia*-like fungi are ecologically hyper-diverse. PLoS Pathog. 2012;10(8): e1004277 10.1371/journal.ppat.1004277.g001 PMC4140847 25144294

[19] Richards TA, Jones MD, Leonard G, Bass D. Marine fungi: their ecology and molecular diversity. Ann Rev Mar Sci. 2012;4: 495–522. 10.1146/annurev-marine-120710-100802 22457985

[20] Ashbee HR. Update on the genus *Malassezia*. Med Mycol. 2007;45(4): 287–303. 10.1080/13693780701191373 . 17510854

[21] Ashbee HR, Evans EG. Immunology of diseases associated with *Malassezia* species. Clin Microbiol Rev. 2002;15(1): 21–57. 10.1128/CMR.15.1.21-57.2002 11781265 PMC118058

[22] Midgley G. Morphological variation in *Malassezia* and its significance in pityriasis versicolor In: Bossche H, Odds FC, Kerridge D, editors. Dimorphic fungi in biology and medicine. Boston, MA: Springer; 1993 p. 267–77.

[23] Kirkland TN, Fierer J. *Coccidioides immitis* and *posadasii*; a review of their biology, genomics, pathogenesis, and host immunity. Virulence. 2018;9(1): 1426–35. 10.1080/21505594.2018.1509667 30179067 PMC6141143

[24] Del Rocio Reyes-Montes M, Perez-Huitron MA, Ocana-Monroy JL, Frias-De-Leon MG, Martinez-Herrera E, Arenas R, et al The habitat of *Coccidioides* spp. and the role of animals as reservoirs and disseminators in nature. BMC Infect Dis. 2016;16(1): 550 10.1186/s12879-016-1902-7 27724885 PMC5057265

[25] Saubolle MA, McKellar PP, Sussland D. Epidemiologic, clinical, and diagnostic aspects of coccidioidomycosis. J Clin Microbiol. 2007;45(1): 26–30. 10.1128/JCM.02230-06 PubMed Central PMCID: PMC1828958. 17108067 PMC1828958

[26] Nguyen C, Barker BM, Hoover S, Nix DE, Ampel NM, Frelinger JA, et al Recent advances in our understanding of the environmental, epidemiological, immunological, and clinical dimensions of coccidioidomycosis. Clin Microbiol Rev. 2013;26(3): 505–25. 10.1128/CMR.00005-13 PubMed Central PMCID: PMC3719491. 23824371 PMC3719491

[27] Munoz-Hernandez B, Martinez-Rivera MA, Palma-Cortes G, Manjarrez E. Innovation of the parasitic cycle of *Coccidioides* spp In: Shah MM, editor. Parasitology: InTech; 2012.

[28] Munoz-Hernandez B, Palma-Cortes G, Cabello-Gutierrez C, Martinez-Rivera MA. Parasitic polymorpism of *Coccidioides* spp. BMC Infect. 2014;14: 213. 10.1186/1471-2334-14-213 PMC4009063 24750998

[29] Kwon-Chung KJ, Fraser JA, Doering TL, Wang Z, Janbon G, Idnurm A, et al *Cryptococcus neoformans* and *Cryptococcus gattii*, the etiologic agents of cryptococcosis. Cold Spring Harb Perspect Med. 2014;4(7): a019760 10.1101/cshperspect.a019760 PubMed Central PMCID: PMC4066639. 24985132 PMC4066639

[30] Steenbergen JN, Shuman HA, Casadevall A. *Cryptococcus neoformans* interactions with amoebae suggest an explanation for its virulence and intracellular pathogenic strategy in macrophages. Proc Natl Acad Sci USA. 2001;98(26): 15245–50. 10.1073/pnas.261418798 11742090 PMC65014

[31] O'Meara TR, Alspaugh JA. The *Cryptococcus neoformans* capsule: a sword and a shield. Clin Microbiol Rev. 2012;25(3): 387–408. 10.1128/CMR.00001-12 PubMed Central PMCID: PMC3416491. 22763631 PMC3416491

[32] Zaragoza O. Basic principles of the virulence of *Cryptococcus*. Virulence. 2019;10(1): 490–501. 10.1080/21505594.2019.1614383 PubMed Central PMCID: PMC6550552. 31119976 PMC6550552

[33] Zaragoza O, Nielsen K. Titan cells in *Cryptococcus neoformans*: cells with a giant impact. Curr Opin Microbiol. 2013;16(4): 409–13. 10.1016/j.mib.2013.03.006 PubMed Central PMCID: PMC3723695. 23588027 PMC3723695

[34] Okagaki LH, Nielsen K. Titan cells confer protection from phagocytosis in *Cryptococcus neoformans* infections. Eukaryot Cell. 2012;11(6): 820–6. 10.1128/EC.00121-12 PubMed Central PMCID: PMC3370461. 22544904 PMC3370461

[35] Gerstein AC, Fu MS, Mukaremera L, Li Z, Ormerod KL, Fraser JA, et al Polyploid titan cells produce haploid and aneuploid progeny to promote stress adaptation. mBio. 2015;6(5): e01340–15. 10.1128/mBio.01340-15 PubMed Central PMCID: PMC4620463. 26463162 PMC4620463

[36] Zaragoza O. Multiple disguises for the same party: the concepts of morphogenesis and phenotypic variations in *Cryptococcus neoformans*. Front Microbiol. 2011;2: 181 10.3389/fmicb.2011.00181 PubMed Central PMCID: PMC3167222. 21922016 PMC3167222

[37] Feldmesser M, Kress Y, Casadevall A. Dynamic changes in the morphology of *Cryptococcus neoformans* during murine pulmonary infection. Microbiology. 2001;147(8): 2355–65. 10.1099/00221287-147-8-2355 11496012

[38] Fernandes KE, Brockway A, Haverkamp M, Cuomo CA, van Ogtrop F, Perfect JR, et al Phenotypic variability correlates with clinical outcome in *Cryptococcus* isolates obtained from Botswanan HIV/AIDS patients. mBio. 2018;9(5): e02016–18. 10.1128/mBio.02016-18 PubMed Central PMCID: PMC6199498. 30352938 PMC6199498

[39] Fernandes KE, Dwyer C, Campbell LT, Carter DA. Species in the *Cryptococcus gattii* complex differ in capsule and cell size following growth under capsule-inducing conditions. mSphere. 2016;1(6): e00350–16. 10.1128/mSphere.00350-16 PubMed Central PMCID: PMC5196034. 28066814 PMC5196034

[40] Hewitt SK, Foster DS, Dyer PS, Avery SV. Phenotypic heterogeneity in fungi: importance and methodology. Fungal Biology Reviews. 2016;30(4): 176–84. 10.1016/j.fbr.2016.09.002

